# Gaussian-preserved, non-volatile shape morphing in three-dimensional microstructures for dual-functional electronic devices

**DOI:** 10.1038/s41467-020-20843-4

**Published:** 2021-01-21

**Authors:** Ziao Tian, Borui Xu, Guangchao Wan, Xiaomin Han, Zengfeng Di, Zi Chen, Yongfeng Mei

**Affiliations:** 1 Department of Materials Science, State Key Laboratory of ASIC and Systems, Fudan University, 220 Handan Road, Shanghai, 200433 China; 2 State Key Laboratory of Functional Materials for Informatics, Shanghai Institute of Microsystem and Information Technology, Chinese Academy of Sciences, Shanghai, 200050 China; 3 Thayer School of Engineering, Dartmouth College, Hanover, NH 03755 USA

**Keywords:** Electronic devices, Nanoscience and technology

## Abstract

Motile plant structures such as *Mimosa pudica* leaves, *Impatiens glandulifera* seedpods, and *Dionaea muscipula* leaves exhibit fast nastic movements in a few seconds or less. This motion is stimuli-independent mechanical movement following *theorema egregium* rules. Artificial analogs of tropistic motion in plants are exemplified by shape-morphing systems, which are characterized by high functional robustness and resilience for creating 3D structures. However, all shape-morphing systems developed so far rely exclusively on continuous external stimuli and result in slow response. Here, we report a Gaussian-preserved shape-morphing system to realize ultrafast shape morphing and non-volatile reconfiguration. Relying on the Gaussian-preserved rules, the transformation can be triggered by mechanical or thermal stimuli within a microsecond. Moreover, as localized energy minima are encountered during shape morphing, non-volatile configuration is preserved by geometrically enhanced rigidity. Using this system, we demonstrate a suite of electronic devices that are reconfigurable, and therefore, expand functional diversification.

## Abstract (web summary)

Designing the functional diversification of electronic devices with morphable 3D structures in multistable states remains a challenge. Here, the authors present a Gaussian-preserved shape-morphing system to realize ultrafast shape morphing and non-volatile reconfiguration developing dual-functional electronic devices, such as switch, actuator, and antenna on microscale.

## Introduction

Nastic motion in motile plants^1^, known as a mechanical movement in response to external stimuli, enables the performance of vital tasks such as self-protection (*Mimosa pudica*)^2^, seed dispersal (*Imatiens glandulifera*)^3^, and food acquisition (*Dionaea muscipula*)^4^. Different from tropistic motion^5^, in which growth movement of the biological organism depends on continuous external stimuli, fast geometric changes in nastic motion are stimuli-independent. Such motion follows Gauss’s *theorema egregium* that Gaussian curvature is an intrinsic measure of curvature on a developable plane and keeps as constant without obvious stretching or compression^6–8^. As a result, after triggered by instantaneous stimuli, motile plant structures exhibit fast mechanical movements in a few seconds or less^9^. By mimicking dynamic architectures in nature, a shape-morphing system^10,11^ incorporated with smart materials has emerged to create on-demand 3D microstructures^12,13^, e.g., microrobots^14,15^, metamaterials^16,17^, 4D printing technology^18,19^ and reconfigurable electronics^20,21^. However, like tropistic motion, all of the shape movements in these systems developed so far rely exclusively on external stimuli, and result in a slow response compared to the nastic model. Moreover, artificial morphed shapes created by shape-morphing systems could not be preserved without driving force and their corresponding transformation is volatile after release or being free. Preserved shape morphing not only enables individual devices with non-volatile reconfigurable functions but also offers a new degree of device diversification in the More-than-Moore application^22,23^.

Herein, we apply a Gaussian-preserved mechanism to dynamically morph nanomembranes, referred to as Gaussian-preserved shape-morphing system, for inducing fast, non-volatile, reconfigurable, and reversible structural transformation. In this system, some degree of stretching is locally involved in the nanomembranes by introducing a folding crease, which allows the nanomembranes to morph non-isometrically. Besides, this stretching is only confined to the narrow creased region, while the majority of thin nanomaterials preserve Gaussian curvature which is bending-invariant and remains zero. As a result, using a Gaussian-preserved system, the transformation between different structures can be triggered by mechanical or thermal stimuli within 4.5 μs–2 s, in contrast with a typical transition time of 0.1–10 s for soft reconfigurable structures in the traditional shape-morphing system. Moreover, the Gaussian-preserved shape morphing enhances structural stability and creates an energy barrier leading to a non-volatile reconfiguration. We apply this system to microelectronics areas for solving the challenge of the diversity of applications. This shape-morphing system can add a new degree of freedom to increase the diversification of applications through geometrical changes. Diverse dual-functional electronic devices, such as switch, actuator, and antenna on the microscale, are fabricated to fulfill functional diversification, opening a new route to “More-than-Moore”.

## Results

### Gaussian-preserved shape morphing and non-volatile reconfiguration

**Fig. 1:**
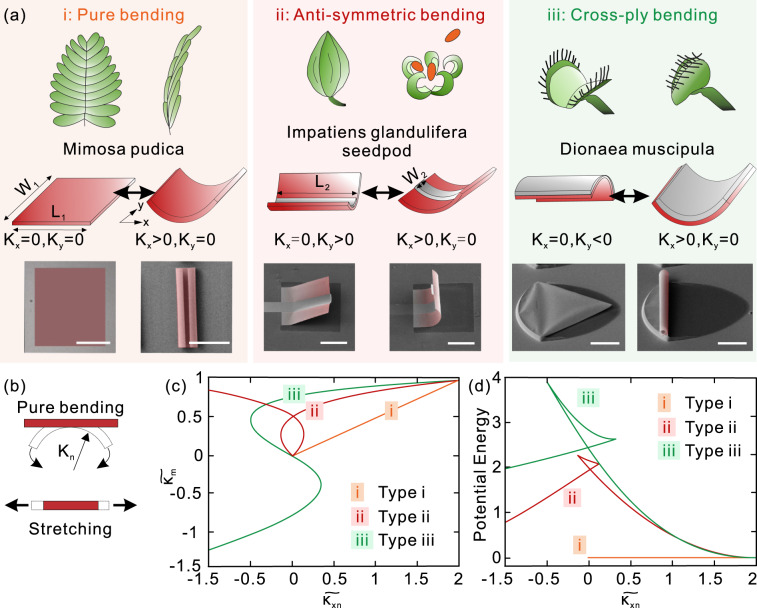
Gaussian-preserved shape morphing in plants and man-made microstructures. **a** A conceptual overview of three types of Gaussian-preserved shape morphing in plants (upper panel), man-made reconfigurable microstructures (middle panel), and corresponding SEM images (lower panel): (i) pure bending, (ii) anti-symmetric bending, and (iii) cross-ply bending. *K*_*x*_ and *K*_*y*_ are the curvatures of nanomembranes in *x*- and *y*-direction, respectively. *W*_*1*_ and *L*_*1*_ are the width and length of nanomembranes, respectively. *W*_2_ and *L*_2_ are the width and length of creases, respectively. The scale bar is 50 μm. **b** The schematic diagram of bending and stretching. *K*_*n*_ is nature curvature. **c** Geometric change with respect to natural curvature for three types of morphing. Orange line, red line, and green line present pure bending type, anti-symmetric bending type, and cross-ply bending type, respectively. It is observed that both type ii and iii situation allows two minimum in the shape-morphing process. **d** Potential energy with respect to natural curvature. The energy barrier of type ii and iii can lead to a non-volatile reconfiguration.

Generally, shape-morphing behavior follows Gaussian-preserved rules, which can be divided into three types as shown in Fig. 1a, named as (i) pure bending, (ii) anti-symmetric bending, and (iii) cross-ply bending. In nature, leaf movement in *Mimosa pudica* is a type (i). As Fig. 1a-i shows, the primary petioles are extended from the stem in a horizontal or ascending direction, and the pinnae are spread out at wide angles with each other. Triggering by extra stimuli, the pinnules fold together in pairs, and the closed pairs point forward towards the tip of the pinna. Type (i) is also generally observed where a flat nanomembrane, of which curvatures in *x*- and *y*-direction are zero (*K*_*x*_ = *K*_*y*_ = 0, left panel in Fig. 1a-i), bends into a curved-up shape. The curved-up shape has one positive curvature *K*_*x*_ > 0, whereas the other one equals zero *K*_*y*_ = 0 (right panel in Fig. 1a-i). In this case, pure bending is mainly driven by the bending energy in nanomembrane, and the contribution of stretching energy is negligble^24^. Many analytical methods were developed to predict the bending results, such as the Timoshenko theory^25^ and history-dependent model^26,27^. Also, the pure bending phenomenon was applied in self-rolled-up nanotechnology with the formation of rolling 3D architectures^28,29^. SEM images in Fig. 1a-i reflects the pure bending of a single-layered Vanadium dioxide (VO_2_) nanomembrane fabricated through the fabrication process in the “Methods” section. Under the strain gradient in the VO_2_ single layer, the nanomembrane bent along *x*-direction resulting in a rolled tubular shape after released from the substrate. The characteristics of VO_2_ nanomembranes are shown in Supplementary Fig. 1.

Different from pure bending, anti-symmetric bending (shown in Fig. 1a-ii) refers to the situation in which curved direction changes from one to the other (*K*_*x*_ > 0 into *K*_*y*_ > 0). According to the Gaussian-preserved rule, the curvature in another direction has to keep at zero as the structure is stabilized (*K*_*y*_ = 0 into *K*_*x*_ = 0). In nature, the seedpod movement of *Impatiens glandulifera* is type (ii). When it is whole, the seedpod is fixed within a shell in a state of tension by connecting adjacent segments. After triggered by extra stimuli, the relaxed shape of a single segment is curled. To realize a man-made anti-symmetric bending, a designed Cr layer with width *W*_2_, length *L*_2_, and thickness *h*_2_ was deposited on VO_2_ nanomembrane with width *W*_1_, length *L*_1_, and thickness *h*_1_. When the parameters were chosen as *W*_1_ = 100 μm, *W*_2_ = 30 μm, *L*_1_ = *L*_2_ = 100 μm, *h*_1_ = 20 nm, and *h*_2_ = 30 nm, Cr/VO_2_ bilayer bent along *x*-direction into a rolled structure due to misfit strain (left panel of Fig. 1a-ii). With a cycle of heating and cooling, VO_2_ nanomembrane undergoes a reversible metal-insulator transition with about 1.07% compressive strain^24^. Notably, instead of returning to the rolled shape, the Cr/VO_2_ bilayer resulted in a folding-like shape. The middle part bent along the transverse direction and a crease-like deformation was generated. After triggered by extra stimuli, the folded shape can morph back to a rolled shape. The whole experimental and simulated shape-morphing processes are shown in Supplementary Movies 1 and 2 and is summarized in Supplementary Fig. 2. It is expected that the existence of two permanent shapes guided by Gaussian-preserved rules makes nanomembrane-based structures useful for reconfigurable devices.

There is another situation that the curvature varies from positive to negative with the change of axis, named as cross-ply bending (Fig. 1a-iii). Leaf movement in *Dionaea muscipula* is type (iii) and is probably one of the best-known rapid motions in the plant kingdom^30^. The trap consists of two lobes attached to the midrib of the organ to form a kind of jaw. The two lobes of the trap are curved outward in the open state and curved inward in the closed state. Upon triggering, the lobes actively change their rest-state curvature in the direction perpendicular to the midrib. In our experiment, the bending of type iii can be observed when the Cr/VO_2_ bilayer nanomembranes have larger *W*_2_. As SEM image presents, nanomembrane curved-up in the *x*-direction or down in *y*-direction as the size of the upper layer is the same as the lower one. And obviously, this phenomenon follows Gaussian-preserved rules, which need further discussion to analytically distinguish cross-ply and anti-symmetric bending.

All stable states in Fig. 1a strictly comply with Gaussian-preserved rules, in which Gaussian curvature keeps zero as the flat nanomembrane does. However, the transformation between these states always requires additional energy to break the rule, known as stretching energy. Considering the size and thickness of nanomembrane, it is expected that the additional energy required to break the rule is much smaller than the plants in nature due to much smaller bending stiffness. As shown in Fig. 1b, bending accounts for the curvature change of deformed nanomembranes and stretching accounts for extension or compression of them. There exists a coupling in bending and stretching mode in the shape-morphing process, which can be characterized through dimensionless elastic energy. The dimensionless elastic energy of nanomembranes with bending and stretching deformation is derived as^4^

1 $$\tilde U = \tilde U_{\mathrm{bending}} + \tilde U_{\mathrm{stretching}} = \left( {\widetilde {\kappa _x} - \widetilde {\kappa _{xn}}} \right)^2 + ({\widetilde {\kappa _y} - \widetilde {\kappa _{yn}}})^2 + \alpha ({\widetilde {\kappa _x}\widetilde {\kappa _y} - 1})^2$$

where $\widetilde {\kappa _x}$ = *κ*_*x*_*/κ*, $\widetilde {\kappa _y}$ = *κ*_*y*_*/κ*, $\widetilde {\kappa _{xn}}$ = *κ*_*xn/*_*κ* and $\widetilde {\kappa _{yn}}$ = *κ*_*yn*_*/κ* are dimensionless parameters representing natural curvature in *x*- and *y*-direction, respectively. Parameter *α* = *W*^4^*κ*^2^/*h*^2^ determines the degree of coupling between bending and stretching. *κ* is the initial curvature. *h* is thickness, and *W* is determined experimentally as *W* = ((*W*_*1*_ + *W*_*2*_)/4). To elucidate the shape-morphing process in Fig. 1a, dimensionless mean curvature ($\widetilde {\kappa _m}$ = ($\widetilde {\kappa _x}{\,}$*+* $\widetilde {\kappa _y}$)/2) was calculated by minimizing the total energy with respect to $\widetilde {\kappa _x}$ and $\widetilde {\kappa _y}$. More details are shown in the “Methods” section. Figure 1c presents the results of $\widetilde {\kappa _m}$ related to dimensionless natural curvature. It is noticed that the route of curvature variation is governed by the value of *α*. In a pure bending situation (*α* = 0), only one minimum is found during the process (orange line in Fig. 1c), thus the shape of nanomembranes changes continuously from planar to curved-up without barrier. As for type ii situation where *W*_2_ = 20 μm and *α* is 0.885, the existence of two minima indicate two stable states (red line in Fig. 1c), corresponding to SEM images in Fig. 1a-ii. When *α* is calculated as 1.335 with *W*_2_ as 100 μm, $\widetilde {\kappa _m}$ passes through zero generating a snap motion (green line in Fig. 1c).

It is observed that both type ii and iii situations allow two minima in the shape-morphing process. To further elucidate their difference, potential energy related to $\widetilde {\kappa _{xn}}$ was carried out as shown in Fig. 1d. For pure bending case, the potential energy keeps zero regardless of $\widetilde {\kappa _{xn}}$ implying a spontaneous shape-morphing process without the need for extra energy. But for the other two cases, the Gaussian-preserved shape morphing enhances structural stability and creates an energy barrier that proclaims the requirement for extra energy to overcome it. Over an energy barrier, the elastic energy can rapidly be converted into kinetic energy, generating a fast motion. We note that the energy barrier becomes bigger as the coupling parameter *α* increases. It means that cross-ply reconfigurable structure needs a larger trigger force than the anti-symmetric reconfigurable structure. In another word, a lower barrier of anti-symmetrical bending offers greater flexibility in design and broader applications with such structure.

### Design strategy of non-volatile reconfiguration structure

**Fig. 2:**
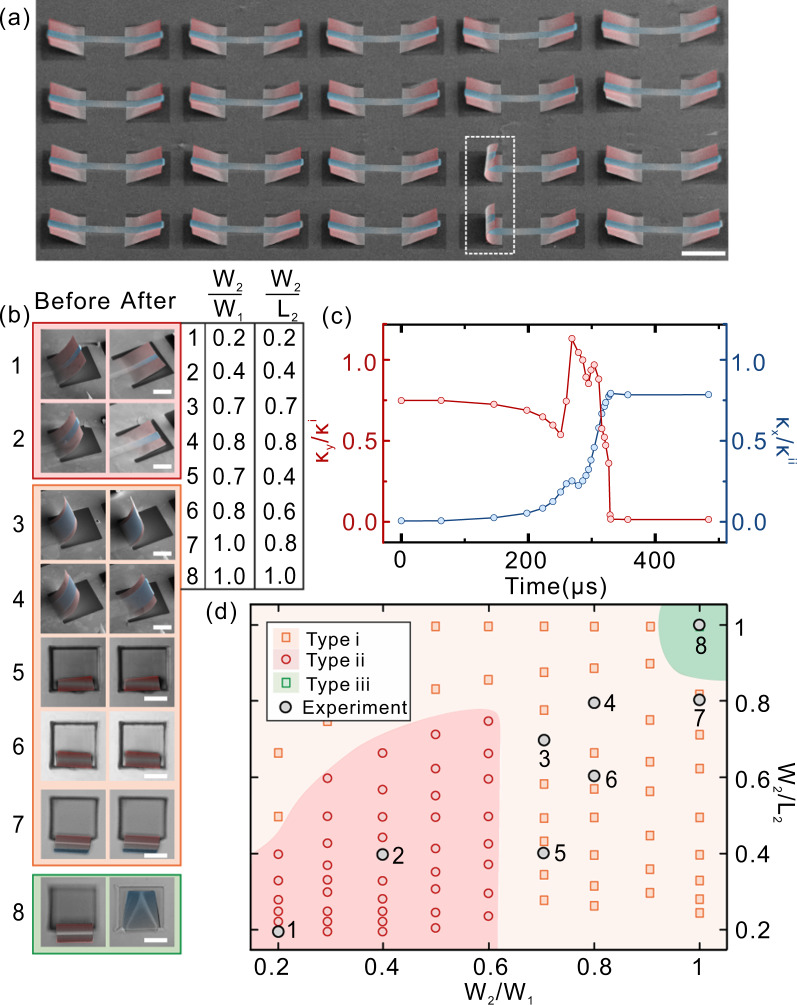
Design strategy for creating non-volatile reconfiguration. **a** SEM image of an array of the curved-up shape with orthogonal curvature through anti-symmetric bending. The white dashed line surrounds two of the folded shape. The scale bar is 100 μm. **b** SEM (1–4) and optical images (5–8) of eight samples before and after shape morphing with geometric parameters shown in the attached table. *W*_2_/*W*_1_ and *W*_2_/*L*_2_ represent the ratio of the upper layer width to lower layer width, and upper layer width to length, respectively. The scale bar is 50 μm. **c** The variations of $\kappa _y/\kappa ^i$ (left axis) and $\kappa _x/\kappa ^{ii}$ (right axis) versus the time from FEM. **d** Phase diagram of favorable shape-morphing status related to *W*_2_/*W*_1_ and *W*_2_/*L*_2_. Gray circles are the results of experiments in **b**. Yellow (pure bending), red (anti-symmetric bending), and green (cross-ply bending) dots are the results from FEM.

As aforementioned, geometric parameters play a crucial role in the determination of shape-morphing type. An array of anti-symmetric bending can be achieved by designing geometric parameters to demonstrate structural reliability as shown in Fig. 2a. Specifically, two nanomembranes form into rolled shape as marked by white dash lines, while others are folded and aligned in a highly ordered manner. To find out the relation between geometry and morphing behavior, we defined two dimensionless parameters as *W*_2_/*W*_1_ and *W*_*2*_/*L*_*2*_ to seek morphing behavior under different values. Figure 2b shows the SEM or optical images of eight samples before and after shape morphing with various *W*_2_/*W*_1_ and *W*_2_/*L*_2_ (parameters are summarized in the right table). The morphing type of sample 1 (*W*_2_/*W*_1_ = 0.2, *W*_2_/*L*_2_ = 0.2) and 2 (*W*_2_/*W*_1_ = 0.4, *W*_2_/*L*_2_ = 0.4) is anti-symmetric bending which is able to form two stable shapes. As *W*_2_/*W*_1_ and *W*_2_/*L*_2_ increase with rising W_2_, the morphing behavior of nanomembrane turns into pure bending from anti-symmetric bending (samples 2–7). Furthermore, with the proper design of parameters (*W*_2_/*W*_1_ = 1 and *W*_2_/*L*_2_ = 1, sample 8), cross-ply bending with bistability is obtained as we predicted in Fig. 1a.

To better understand the relation between geometry and morphing behavior, we output the analytical distribution of *κ*_*x*_ and *κ*_*y*_ during the shape morphing between two shapes as shown in Fig. 2c. *κ*_*x*_ and *κ*_*y*_ are the bending curvature along the longitudinal and transverse direction, respectively. For the folded shape, $\kappa _y \approx \left( {1 + \nu } \right)\kappa ^{i}$, $\kappa _x \approx 0$ (*v* is the Poisson’s ratio, more details of .. can be found in “Methods” of FEM part) when the time *t* starts (*t* = 0). When the folded shape morphs to a rolled shape, *κ*_*x*_ abruptly increases and approaches the asymptotic value of $\kappa ^{ii}$ whereas *κ*_*y*_ gradually decreases to zero, suggesting that the Gaussian curvature keeps zero before and after shape morphing. Interestingly, in the shape-morphing process, there is a doubly curved region implying that stretching is involved in the nanomaterials locally by introducing a folding crease. We also output the distributions of $\kappa _x$, $\kappa _y$, the Gaussian curvature $K = \kappa _x\kappa _y$, and strain energy density *u*, along the centerline (*y* = *0*) with respect to the distance *x* in both shapes as shown in Supplementary Fig. 3. It is found that the “folding crease” indeed concentrates strain energy, which results in the energy barrier necessary for bistability. This phenomenon is similar to the occurrence of localized creases or vertices when a thin film cannot deform isometrically under certain geometric constraints. It also implies that the elastic energy significantly decreases and gets converted into kinetic energy, which can speed up the shape transition.

A more detailed investigation of the morphing behavior related to geometric parameters was carried out through a large number of simulations, and analytical results were plotted in the phase diagram of Fig. 2d, together with experimental results above. Selected FEM results with different geometry parameters are shown in Supplementary Fig. 4a. The diagram is divided into three regions according to a different type of morphing behavior. When *W*_2_/*W*_1_ is smaller than 0.7, the anti-symmetric type morphing behavior is obtained with small *W*_2_/*L*_2_. And the bistability of nanomembrane is broken with the increasing value of *W*_2_/*L*_2_. This can be attributed to the requirement for the length of *L*_2_ to permit a smooth exchange in the value of curvature in *x-* and *y*-direction with attainable stretching energy (double curvature in Fig. 2c). As the value of *W*_2_/*W*_1_ rises more than 0.7, anti-symmetric type morphing is extinct, mainly owing to the difficulty in the formation of “folding crease” with a wider upper layer. Besides, it is noteworthy that, with *W*_2_/*W*_1_ = 1 and *W*_2_/*L*_2_ = 1, the morphing behavior of nanomembrane turns into a cross-ply type with bistability, as the width of nanomembrane is big enough to support the bending along the *y* axis. Moreover, as we plotted experimental results into this diagram, the regions they located fit well with their actual morphing behavior. Here, considering that the bistability of nanomembrane is established on Gaussian-preserved situation, the result in this diagram can be extended to other material systems and different scales. We also plot the phase diagram of the through-the-thickness geometry (Supplementary Fig. 4b) to illustrate that the bistability depends on the geometric parameters of the nanomembrane’s planform instead of the through-the-thickness geometry.

### Ultra-rapid dynamic characteristic of Gaussian-preserved shape morphing

**Fig. 3:**
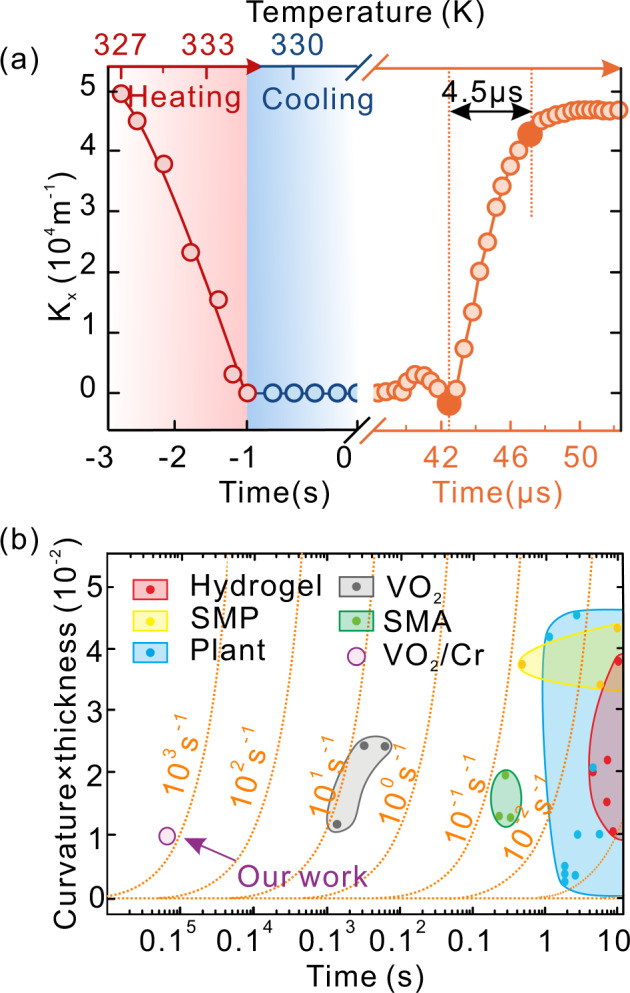
Dynamic characteristic of Gaussian-preserved shape morphing. **a** Curvature in *x*-direction as a function of temperature varied with time. Red and blue circles present heating and cooling processes, respectively, indicating the shape-shifting process from the rolled shape (*K*_*x*_ = 5 × 10^4^ m^−1^) to the folded shape (*K*_*x*_ = 0). The orange circles present the shape-shifting process within 4.5 μs from folded shape to rolled shape triggered by mechanical stimuli. **b** Actuation speed, orange dashed line, and relative curvature change of membrane-based actuators in the literature and our work.

Gaussian-preserved shape morphing enhances structural stability and creates an energy barrier that leads to a non-volatile folded shape. In another word, owing to the breakage of the Gaussian-preserved rule with external energy input, the folded shape loses structural stability and transfers to a rolled shape. The process of anti-symmetric bending was revealed experimentally through the variation of curvature related to time, as shown in Fig. 3a. Changing temperature from 327 to 335 K and back to 327 K in 3 s, rolled shape (curvature in the *x*-direction, *K*_*x*_ is about 5 × 10^4^ m^−1^) can transfer to folded shape (*K*_*x*_ = 0) utilizing the metal-insulator phase transition of VO_2_^20,31^. In this process, the time of Gaussian-preserved shape morphing mainly depends on the heating and cooling time and is limited by the diffusion of heat in VO_2_. We refer to this thermal-triggered shape morphing as slow morphing.

Reversely, the shape transition from folded shape to rolled shape requires additional stretching energy to break the Gaussian-preservation. Here, we use a mechanical probe, as an optional method, to trigger this morphing. When the probe reached a critical position, the current folded shape lost stability. As a result, the structure jumped to the other stable shape, as rolled shape, accompanied by the quick conversion of stored elastic energy to kinetic energy. Such a process is typically fast and only requires a period of as short as 4.5 μs in our case, which is much shorter than the first morphing. We refer to this mechanical-triggered shape morphing as fast morphing. As a good approximation, the timescale of the elastic snap-through can be estimated as $t\sim L^2\sqrt {\rho /E}$ = 4.3 μs (*L* ≈ 30 μm is the characteristic length scale, *E* ≈ 25 GPa is Young’s modulus and *ρ* ≈ 7.19 g/cm^3^ is the mass density)^32^. Since our morphing structures are made of rigid materials with high Young’s modulus on the microscale, this expression suggests that the snapping time can be much shorter than those in polymeric actuators^33,34^. Notably, this conjunction of two significantly different timescales in our case is also found in nature such as the snapping of Venus flytrap (*Donaea muscipula*)^30,35^, the sucking of carnivorous bladderwort (*Utricularia australis*)^36^ and the ejection of the sporangium of *Polypodium aureum*^37^. Inspired by the sporangium’s ejection, we designed an artificial micro-catapult to realize energy storage and release (displayed in Supplementary Fig. 5).

The actuation speed of our work compared to the membrane actuators in the literature is shown in Fig. 3b. Since the bending curvature scales inversely with the membrane thickness, we plot “curvature × membrane thickness” versus time. The constant actuation speed, curvature × thickness/time, is represented as orange lines in Fig. 3b. It’s noticed that the speed of non-volatile shape in our case can be six orders of magnitude higher than the shape-memory polymer (SMP)-based actuators, hydrogel-based actuators, VO_2_-based actuators, and plants, and five orders of magnitude higher than shape-memory-alloy (SMA)-based actuators. The reason behind that is our design followed by the Gaussian-preserved rule, which establishes two stable states with a rapid transition between them rather than a smooth motion during actuation.

### Designability of flexible structure with non-volatile reconfiguration

**Fig. 4:**
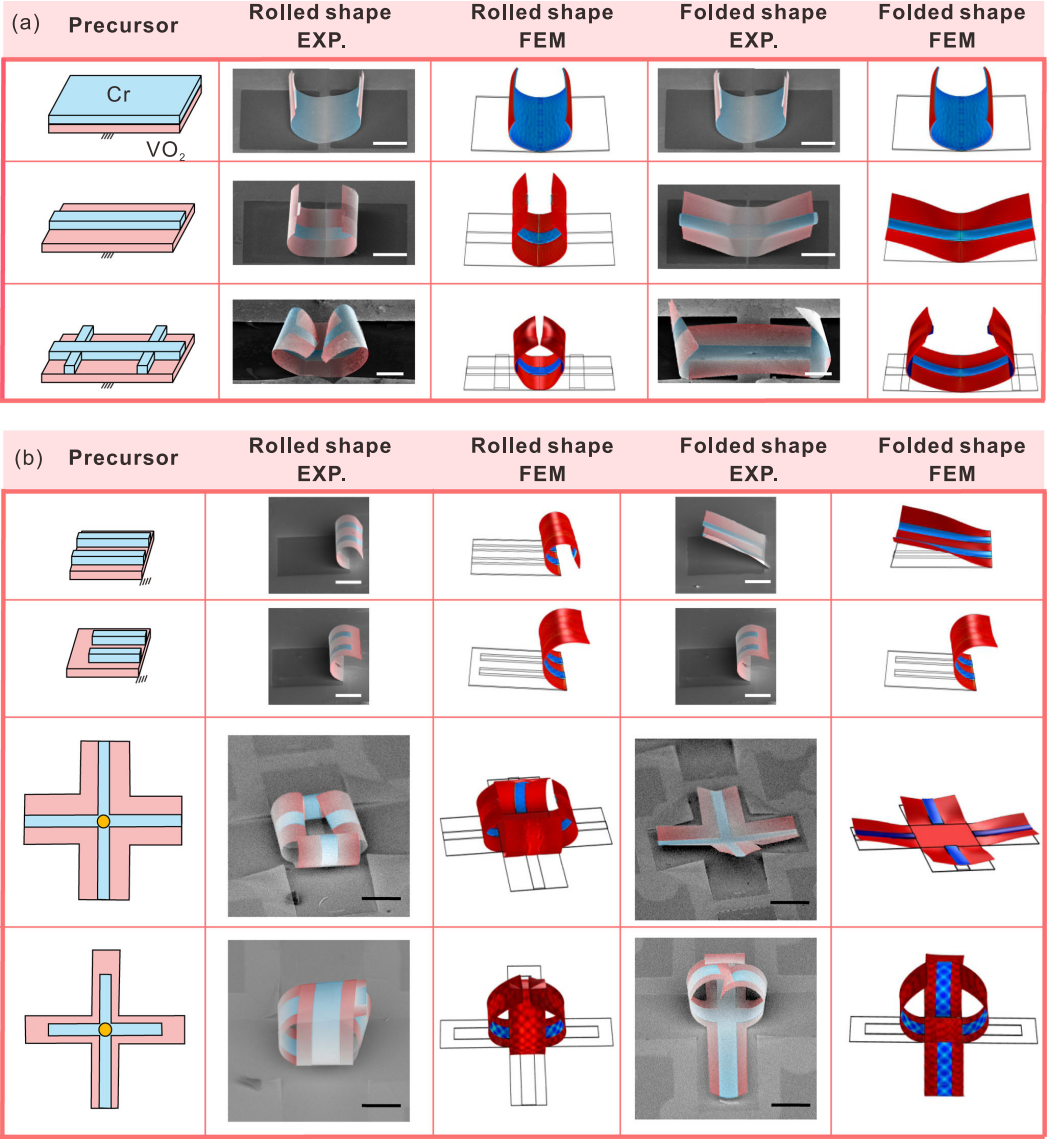
Various reconfigurable 3D microstructures based on non-volatile shape morphing. **a** Design scheme, SEM images, and simulations of Cr/VO_2_ bimorph with different width and arrangement of Cr ribbons. Oblique lines in precursors refer to the bonding position with the substrate. The scale bar is 50 μm. **b** Design scheme, SEM images, and simulations of Cr/VO_2_ bimorph with reprogrammable creases. The first two rows are the design of single-arm with different Cr ribbons tuning bistable states. The third and fourth rows are symmetric and asymmetric four-arm grabbing actuators. Yellow circles in precursors refer to the bonding position with the substrate. The scale bar is 40 μm.

As discussed above, the non-volatile reconfiguration of a single Cr/VO_2_ nanomembrane is primarily determined by its geometry. Therefore, configurations of assembled microstructures can also be tuned by deliberately tailoring the geometry of their 2D precursors to achieve complex behaviors. As a simple start, we first clamped two bistable units to the same edge. When Cr and VO_2_ layers have the same width, the unit is monostable and can only bend along the longitudinal direction (Fig. 4a, first row). By decreasing the width of the Cr layer, either unit can adopt two shapes and the assembled microstructure can become a “two-armed gripper” or a “flapping bird” (Fig. 4a, second row). We can also produce a new “clamped edge” inside each unit by additionally depositing a narrow Cr strip along the transverse direction. As a result, the nanomembrane was divided into two regions. Only the region between the clamped edge and the transverse Cr strip switched between two stable shapes, while the region near the tip only rolled-up along the longitudinal direction (Fig. 4a, third row).

Likewise, instead of being bonded along the longitudinal direction, two units can also be bonded side by side along the transverse direction. This idea can be realized by depositing two parallel Cr strips on one VO_2_ layer (Fig. 4b, first and second row). It is found that this assembled microstructure shared similar bistable behavior with the unit, that is, the folded shape will lose stability when the length of the Cr layer decreases, due to the difficulty in the formation of the folding crease with a wider layer. These design ideas can be further extended by assembling more units together. Here, we bonded four Cr/VO_2_ units to the four sides of a square SiO_2_ substrate. If four units are all bistable, then the assembled microstructure can work as a “four-armed gripper” (Fig. 4b, second row) by grabbing (left) and releasing (right) items when each unit switched between rolled shape and folded shape. Specifically, four arms in folded shapes can automatically curve up in rolled shape to grab objects with mechanical stimuli from the target object (displayed in Supplementary Fig. 6). Such a design can also be modified to add more maneuverability. As shown in the fourth row of Fig. 4b, if we replace three bistable units with monostable units by decreasing the lengths of their Cr layers, only one left bistable unit switched to stable folded shape and became flat to enable the releasing of carried objects. This allows us to control the direction of cargo release. Moreover, in all cases, FEM predictions agree well with experimental observations except the last one, which is attributed to the contact during deformation in a defined symmetric pattern. One Gaussian-preserved shape-morphing unit can take two stable states. If we put *N* Gaussian-preserved units together, ideally, the assembled microstructure can have 2^*N*^ configurations at most. Based on this idea, we show that diverse sets of 3D microstructures can be assembled by appropriately combining several Cr/VO_2_ bistable or monostable units together.

### Dual-functional architecture devices

**Fig. 5:**
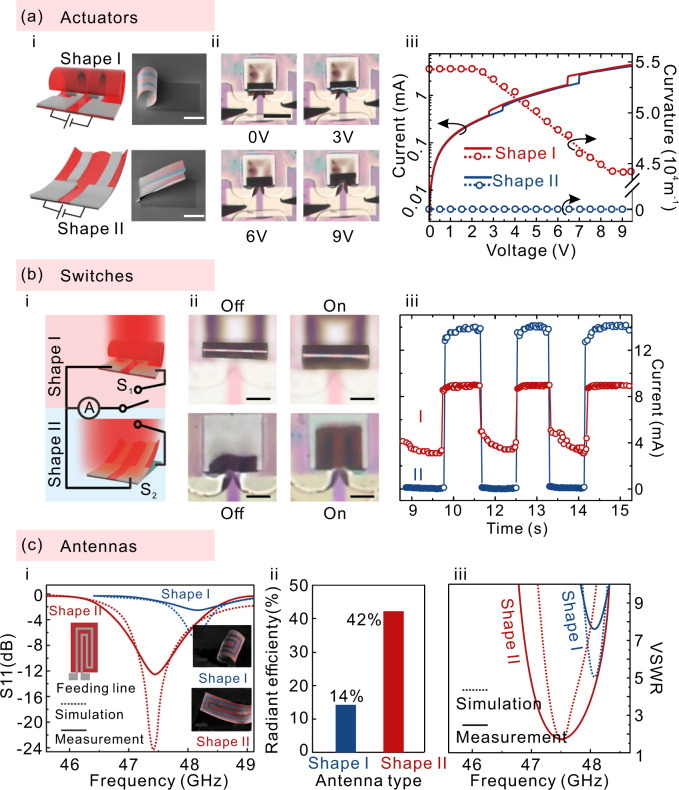
Dual-functional architecture devices. **a** MEMS microactuators controlled by electric signals. (i) Schematics and SEM images of microactuators. The scale bar is 40 μm. (ii) Optical images of actuating behavior of shape I actuator with various voltages. The scale bar is 100 μm. (iii) Current–voltage characteristics (solid line) and corresponding curvature (dashed line) change of two shapes. Empty circles present experimental curvature. **b** MEMS switches. (i) Schematic of morphable MEMS switches. The shape I and II serve as a different type of switches (S_1_ and S_2_), which can turn into the other with extra stimuli. (ii) Optical images of the switches in shape I (upper) and II (lower) without (off) and with laser irradiation (on). The scale bar is 30 μm. (iii) Current change with laser irradiation as the on-off of the switch. The red and blue lines represent the switch in shape I and II, respectively. **c** Concealable antennas. (i) Measured (solid line) and simulated (dashed line) return loss (S11) versus frequency for the shape I and II, respectively. Left inset is the scheme of 2D precursor. Top-right and bottom-right insets show the SEM images of antennas in shape I and II, respectively. The scale bar is 100 μm. (ii) Simulated radiant efficiencies of the antenna type. (iii) Measured and simulated VSWR versus frequency response for two antennas.

Unlike More Moore, More-than-Moore has not emphasized miniaturization, but the trend for increased functional diversification. To this end, extremely diverse applications have been fabricated as well as a diversity of materials and methods have been applied to manufacture them. Due to the one-device one-design paradigm of discrete technologies, each device had a unique design and a unique manufacturing process. Predictably, still continuing to provide ever-increasing functionality, the manufacturing cost of applications tends to be higher. For all of these reasons, a major challenge to electronic technology has been the diversity of applications. The capability to both shape and property reconfigurations allows us to achieve diverse electronic devices as shown in Fig. 5. Combining the electrical characteristic of phase transition in VO_2_ and reconfigurable structures, dual-functional MEMS actuators were fabricated. The two-terminal devices, shown schematically in Fig. 5a, are composed of two Cr electrodes on VO_2_ nanomembrane, where the electrode was designed with the width in 10 μm separated by a gap in 20 μm. Two shapes (rolled shape refers to shape I and folded shape refers to shape II) can be both formed and SEM images are shown in Fig. 5a-i. The device can be electrically actuated by Joule heating of flowing current through itself. The actuating behavior of shape I with various voltages are shown in Fig. 5a-ii and Supplementary Movie 3. With the rising of voltage from 0 V to 9 V, the curvature of shape I decreases from 5.5 to 4.4 × 10^4^ m^−1^, while it keeps zero for shape II, as shown in Fig. 5a-iii. In this figure, some interesting phenomena are also observed from the current–voltage (*I*–*V*) transfer characteristics of the two devices. Firstly, all devices exhibited a conductance switching phenomenon where there is a sharp rise in current, reflecting the fast transition of VO_2_ when the voltage was raised above the threshold point (*V*_ON_, *I*_ON_). There exists a nonlinear *I*–*V* behavior due to the non-fixed VO_2_/Cr structure. Secondly, *V*_ON_ of shape I is monotonically lower than folded shape, suggesting that the transition temperature of shape II is lower than the one of folded shape. This is due that as the curvature of shape I is larger, VO_2_ nanomembrane in rolled shape generates larger additional compressive strains, resulting in lower transition temperature^20^. It implies that Gaussian-preserved shape morphing could lead a new family in the material with novel properties. Finally, there are two conductance switching phenomena with increasing voltage. The lower one is attributed to the transition that happened between the electrodes, while the other rise owes to the complete transition in VO_2_ nanomembrane. An array of actuators and simulated temperature distribution are shown in Supplementary Fig. 7.

Dual-functional MEMS switches based on bistable Cr/VO_2_ nanomembranes were fabricated as shown in Fig. 5b. Instead of a steady current supply, we replaced the joule heating with a focused infrared laser at 808 nm with a spot size of ~1.5 μm to apply a periodic heat stimulus to both stable shapes. The experimental and simulated response frequencies of both stable shapes are shown in Supplementary Fig. 8. The response of folded shape is faster than rolled shape, indicating that the actuation performance of our structure is more sensitive to the change of its working environment. The regulation mechanism of the first function is based on the electrical resistance’s change of VO_2_ during the phase transition (upper panels in Fig. 5b-i, ii). When irradiated by laser, the temperature of the Cr/VO_2_ nanomembrane increased and hence lowered the electrical resistance. Correspondingly, the curvature of the shape I decreased (Supplementary Movie 4), and the circuit’s current increased. MEMS switches with different curvatures were also fabricated (Supplementary Fig. 9). The second function of the MEMS switch took the form of shape II and relied on physical contact to make the circuit open or closed (lower panel in Fig. 5b-i, ii). When laser irradiation was applied on the shape II device, VO_2_/Cr bimorph got flatten and contact with the circuit, which is similar to the isolating switch in the form of a suspended micro-cantilever (Supplementary Movie 5). The responses of two types of MEMS switches under light stimuli were tested by sequentially turning on and off the laser irradiation (Fig. 5b-iii). When the laser was turned on, the current decreased in both cases. For the shape I device, the current decreased from ~9 to ~3 mA due to the phase transition of VO_2_. A more drastic current decrease from 14 to 0 mA was observed in the shape II device because of the open circuit. The difference in current change between these two types of switches suggests that our optically activated switches can regulate the electrical circuit with more maneuverability.

Another MEMS device, a concealable antenna was demonstrated as shown in Fig. 5c with a 2D precursor shown in the left inset. An antenna can transmit electromagnetic waves to space from conductive components. The property of an antenna highly depends on its physical geometry. The working frequency of the concealable antenna will shift as a result of mechanical deformation. Figure 5c-i presents the measured and simulated spectrum responses of the reflection coefficient (S11) of the antenna at two different structural states over a frequency range of 46–50 GHz (The electrical connecting between antenna and SubMiniature version A connectors are shown in Supplementary Fig. 10). The S11 curve exhibits a leftward shift from shape I to shape II due to the change in total capacitance of effective radiating elements. With morphing shape from II to I, working frequency shifts from 47.4 to 48 GHz and S11 falls below −4 dB. The radiant efficiencies obtained by simulation (Fig. 5c-ii) show that shape I (14%) has a much smaller radiant efficiency than shape II (42%), due that the metallic coil is exposed. The antenna performance has also been quantitatively evaluated by testing of the voltage standing wave ratio (VSWR), which is a unitless ratio ranging from 1 to infinity expressing the amount of reflected energy at the input of the device. The simulation and measurement results of VSWR are shown in Fig. 5c-iii, where good agreements can be observed. Compared to shape I, the VSWR of shape II is closer to 1, meaning that impedance-matching of the antenna is closer to an ideal value. The measured center frequencies of shape I and shape II are 48.1 and 47.5 GHz with a 5.83:1 bandwidth of 0 and 1.3 GHz (more details see Supplementary Fig. 10), respectively. The radiant quality factor is then calculated as 0 and 3.89 for shape I and shape II, respectively. These results suggest that shape II is a working model, where the antennas are elevated and exposed. After shape morphing from shape II to shape I, the VO_2_ covers the coil, such that it is electromagnetically shielded. A ribbon-like antenna was also designed to decrease the working frequency as shown in Supplementary Fig. 11.

## Discussion

In summary, Gaussian-preserved shape morphing is the perfect route to markedly increase maneuverability and enrich the design space of microstructures. These microstructures, such as “two-armed gripper” and “flapping bird”, can be precisely tuned and preserved by tailoring the 2D precursor’s geometry relying on Gaussian-preserved principle regardless of the constituent materials and the length scale. This concept can be applied in microelectronics to expand the functional diversification of electronic devices, with morphable 3D structures in multistable states. Offering structurally creasing nanomembranes, diverse dual-functional electronic devices can be readily switched using extra stimuli. Our method not only achieves the generation of non-volatile shape morphing in nanomembranes through exploiting the Gaussian-preserved principle for the first time but also provides a manufacturing paradigm of diversification for the “More-than-Moore” roadmap.

## Methods

### VO_2_ nanomembranes growth

VO_2_ thin films were grown on SiO_2_ coated Si substrates using a radio frequency magnetron sputtering of a V_2_O_5_ target. The growth temperature, growth pressure, and Ar gas flow were 550 °C, 10 mTorr, and 90 sccm, respectively.

### Fabrication of rolled-up shape

All of the samples were patterned by photolithography and reactive ion etching (RIE). Firstly, the photoresist, AZ-5214 (Microchemicals GmbH, Germany), with about 1 μm thickness was spin-cast and photolithography patterned on the VO_2_ as an etching window pattern. Then, the patterned sample was etched by RIE under the following conditions: 15 sccm CF_4_ flow rate, 30 sccm Ar flow rate, 300 mT chamber pressure, and 100 W power for 100 s. A thinning VO_2_ was fabricated by a second photolithography step. The designed thinning part was then etched by RIE under the following conditions: 10 sccm CF_4_ flow rate, 30 sccm Ar flow rate, 100 mT chamber pressure, and 80 W power for 15 s. A narrow strip of Cr with the thickness 30 nm was deposited on thinned VO_2_ by electron beam evaporation method. After that, 40% HF (hydrofluoric acid) solution was used to selectively remove SiO_2_ layer to undercut and release the nanomembranes without damaging. The etching rate was around 10 nm/min. Finally, critical point drying was applied to dry the rolled-up nanomembranes without structural collapse.

### Characterization of morphology and actuating behavior

The morphology of microactuators was observed by scanning electron microscope (Field Emission SEM, Zeiss) and transmission electron microscopy (TEM, Zeiss). The temperature of the sample was controlled by a hot plate with a liquid nitrogen cooling system (THMS600, Linkam). The heating and freezing rates were 0.01 K/s. At the same time, the shape-changing of the microactuator was observed using an optical microscope (BX51, Olympus) and recorded by the camera at 30 fps (FR180, Norpix).

### Finite element method of bistable structures

The geometry model is illustrated in Supplementary Fig. 3. Hypothetical thermal expansion coefficients are assigned to both materials because the mismatch strain between two layers is conceptually generated from different thermal expansions in two layers, which can be simulated by applying a thermal load on either one of the two layers. The Young’s modulus and Poisson’s ratio of Cr and VO_2_ are chosen as *E*_2_ = 25 GPa, *E*_1_ = 86 GPa, *v*_2_ = 0.2, *v*_1_ = 0.3^38–40^. The subscript “1” and “2” denote to properties of VO_2_ and Cr, respectively. The misfit strain is reproduced in simulations through the thermal strain, and we assign the thermal expansion coefficient to both layers as 0.001. As for the thermal distribution, the parameters of electrical conductivity for VO_2_ layer in the metal phase and insulator phase were set as 6 × 10^5^ and 8 × 10^2^ S/m, respectively. To reproduce the clamped boundary condition, the cross-section of one end was set fixed that allows no displacement and rotation. Each cell was constructed of 20-node quadratic brick (C3D20) and mesh accuracy was verified through the refinement study. The general static step was chosen since we seek the stable, equilibrium shape and the nonlinear geometry option was activated. In the dynamic process, a rigid hemisphere moved along the *x*-direction and pushed the Cr/VO_2_ thin film towards the clamped end until it snapped (Supplementary Movie 2). The interaction between the hemisphere and the bilayer Cr/VO_2_ was set as hard contact without friction. Besides, during the snap-through, the energy that was dissipated by viscous damping was kept below 0.1% of the total energy. Since the deformation of the bimorph contains mirror-symmetry with respect to the plane *y* = 0, we only performed simulation on half of the structure and applied the symmetry boundary condition on the plane *y* = 0 to reduce the computational cost.

As mentioned in the main context, we introduce the intrinsic curvature $\kappa ^i$ of the middle Cr/VO_2_ part to rescale the distribution of $\kappa _x$ and $\kappa _y$ in both shapes. Unlike the actual bending curvature, the intrinsic curvature is defined as the curvature that can make the thin structure locally stress-free^6^. In our case, it can be understood as the bending curvature of an extremely narrow strip of Cr/VO_2_ bimorph driven by the equal biaxial misfit strain $\varepsilon _m$. As a quantitative measurement, we used the expression from Timoshenko^25^ as

2 $$\kappa ^i = \frac{{6mn\left( {n + 1} \right)^2}}{{m^2n^4 + 1 + 4mn\left( {n^2 + 1} \right) + 6mn^2}}\frac{{\varepsilon _m}}{{h_1 + h_2}}$$

where $m = E_1/E_2$ and $n = h_1/h_2$ are the ratio of Young’s modulus and thickness between the Cr and VO_2_ layers, respectively. we revised $\kappa ^i$ by considering the bending of the monolayer VO_2_ and introduced the “average” intrinsic curvature $\kappa ^{ii}$ to rescale $\kappa _x$

3 $$\kappa ^{ii} = \frac{{6mn\left( {n + 1} \right)^2}}{{\frac{W_2}{W_1}m^2n^4 + \frac{W_1}{W_2} + 4mn\left( {n^2 + 1} \right) + 6mn^2}}\frac{{\varepsilon _m}}{{h_1 + h_2}}$$

Since the intrinsic curvature of the monolayer VO_2_ is zero, it is not appropriate to use the intrinsic curvature $\kappa ^i$ of the middle part alone to rescale the curvature $\kappa _x$.

Elastic plate statics. The bending energy of the plate is:

4 $$U_{\mathrm{bending}} = \frac{{Eh^3L^2}}{{24\left( {1 - \nu ^2} \right)}}\left[ {\left( {\kappa _x - \kappa _x^i} \right)^2 + ({\kappa _y - \kappa _y^i})^2 +{\,} 2\nu ({\kappa _x - \kappa _x^i})({\kappa _y - \kappa _y^i}) + 2({1 - \nu })({\kappa _{xy} - \kappa _{xy}^i})^2} \right]$$

The subscript *i* denotes the intrinsic curvature. For the equally biaxial misfit strain, the intrinsic curvatures are $\kappa _x^i = \kappa _y^i = \kappa ^i = 3\varepsilon /2h$, $\kappa _{xy}^i = 0$. These expressions are based on the presumption that the two layers are the same. If the top and bottom layers have different materials or thickness, the expression will be different. However, it will not affect the bifurcation diagram qualitatively.

The stretching energy, without the introduction of further derivation, can be written as

5 $$U_{\mathrm{stretching}} = \frac{{EhL^6}}{{24{\mathrm{{\Lambda}}}}}({\kappa _x\kappa _y - \kappa _{xy}^2})^2$$

For a square platform, the coefficient ${\mathrm{{\Lambda}}} = 16\pi ^2/0.738$ based on the previous numerical investigation. Therefore, the total, dimensionless elastic energy $\tilde U$ $\left( {U = U_{\mathrm{b}} + U_{\mathrm{s}} = \tilde UEL^2h/24\left( {1 - \nu ^2} \right)} \right)$ can be expressed as

6 $$\tilde U = \left( {\widetilde {\kappa _x} - \widetilde {\kappa ^i}} \right)^2 + \left( {\widetilde {\kappa _y} - \widetilde {\kappa ^i}} \right)^2 +{\,} 2\nu \left( {\widetilde {\kappa _x} - \widetilde {\kappa ^i}} \right)\left( {\widetilde {\kappa _y} - \widetilde {\kappa ^i}} \right) + 2\left( {1 - \nu } \right)\left( {\widetilde {\kappa _{xy}}} \right)^2 + \frac{{\left( {1 - \nu ^2} \right)L^4}}{{{\mathrm{{\Lambda}}}h^4}}\left( {\widetilde {\kappa _x}\widetilde {\kappa _y} - \widetilde {\kappa _{xy}}^2} \right)^2$$

The stable, equilibrium shapes are sought as the minimum potential energy as $\partial \tilde U/\partial \widetilde {\kappa _x} = 0$, $\partial \tilde U/\partial \widetilde {\kappa _y} = 0$ and $\partial \tilde U/\partial \widetilde {\kappa _{xy}} = 0$. Since twisting is absent, we have

$$({\widetilde {\kappa _x} - \widetilde {\kappa ^i}}) + \nu ({\widetilde {\kappa _y} - \widetilde {\kappa ^i}}) + \left( {1 - \nu ^2} \right)\varphi \widetilde {\kappa _x}\widetilde {\kappa _y}^2 = 0$$

7 $$({\widetilde {\kappa _y} - \widetilde {\kappa ^i}}) + \nu ({\widetilde {\kappa _x} - \widetilde {\kappa ^i}}) + \left( {1 - \nu ^2} \right)\varphi \widetilde {\kappa _y}\widetilde {\kappa _x}^2 = 0$$

where $\varphi = L^4/{\mathrm{{\Lambda}}}h^4$. Subtracting the first equation by the second, we have $\left( {\widetilde {\kappa _x} - \widetilde {\kappa _y}} \right)\left[ {1 - \left( {1 + \nu } \right)\varphi \widetilde {\kappa _x}\widetilde {\kappa _y}} \right] = 0$. It can be satisfied by either $\widetilde {\kappa _x} = \widetilde {\kappa _y}$ or $\left( {1 + \nu } \right)\varphi \widetilde {\kappa _x}\widetilde {\kappa _y} = 1$. When the two equations are satisfied simultaneously, it gives the bifurcation threshold as $\widetilde {\kappa ^{ic}} = 2/\sqrt {\left( {1 + \nu } \right)^3\varphi }$, $\widetilde {\kappa ^c} = \left( {1 + \nu } \right)\widetilde {\kappa ^{ic}}/2$. When $\widetilde {\kappa _x} = \widetilde {\kappa _y}$, we have

8 $$\widetilde {\kappa _x} = \widetilde {\kappa _y} = \widetilde {\kappa ^c}\left[ {\root {3} \of {{\frac{1}{{1 - \nu }}\frac{{\widetilde {\kappa ^i}}}{{\widetilde {\kappa ^{ic}}}} + \sqrt {\left( {\frac{1}{{1 - \nu }}\frac{{\widetilde {\kappa ^i}}}{{\widetilde {\kappa ^{ic}}}}} \right)^2 + \frac{1}{{27}}\left( {\frac{{1 + \nu }}{{1 - \nu }}} \right)^3} }} + \root {3} \of {{\frac{1}{{1 - \nu }}\frac{{\widetilde {\kappa ^i}}}{{\widetilde {\kappa ^{ic}}}} - \sqrt {\left( {\frac{1}{{1 - \nu }}\frac{{\widetilde {\kappa ^i}}}{{\widetilde {\kappa ^{ic}}}}} \right)^2 + \frac{1}{{27}}\left( {\frac{{1 + \nu }}{{1 - \nu }}} \right)^3} }}} \right]$$

When $\left( {1 + \nu } \right)\varphi \widetilde {\kappa _x}\widetilde {\kappa _y} = 1$, we have

$$\widetilde {\kappa _x} = \widetilde {\kappa ^c}\left[ {1 \pm \sqrt {1 - \left( {\frac{{\widetilde {\kappa ^{ic}}}}{{\widetilde {\kappa ^i}}}} \right)^2} } \right]$$

9 $$\widetilde {\kappa _y} = \widetilde {\kappa ^c}\left[ {1 \mp \sqrt {1 - \left( {\frac{{s\widetilde {\kappa ^{ic}}}}{{\widetilde {\kappa ^i}}}} \right)^2} } \right]$$

### Testing of electrical and optical activated switches

Both rolled shape and folded shape were triggered by laser with the wavelength at 808 nm and power density of 0.05 mW/μm^2^, which was focused on ~20 μm in diameter on the bimorph surface. Besides, due to the different behavior of rolled shape and folded shape with irradiation, the pins for measuring current were placed in different locations. For rolled shape, they were placed at two Cr electrodes. And for the folded shape, the pins were placed at the electrode and substrate separately.

The sample was placed on top of a two-dimensional translation stage. One light path assists the alignment of laser sand samples, while another light path was used to observe the actuators’ motion with the help of a high magnification lens. For the time response measurement, a high-speed camera (Nikon 1 with 1200 fps) was used for capturing displacement.

During the photothermal actuation, the laser spot was first aligned with actuators to have maximum displacement, then the laser was taken off. Then an external trigger signal (square wave with a range of from 1 to 100 ms pulse width) was connected to the laser current driver to generate short laser pulses.

## Supplementary information

Supplementary Information

Description of Additional Supplementary Files

Supplementary Movie 1

Supplementary Movie 2

Supplementary Movie 3

Supplementary Movie 4

Supplementary Movie 5

## Data Availability

The authors declare that all data supporting the findings of this study are available from the corresponding author on request.
