# Supplementary material for: Gaussian-preserved, non-volatile shape morphing in three-dimensional microstructures for dual-functional electronic devices
Source: Nat Commun. 2021 Jan 21;12:509. doi: 10.1038/s41467-020-20843-4 (PMC7820288; doi:10.1038/s41467-020-20843-4)
Supplement: Supplementary file 1 — Supplementary Information [file 41467_2020_20843_MOESM1_ESM.pdf]

Supplementary Information

Gaussian-Preserved, Non-Volatile Shape Morphing in Three-Dimensional Microstructures for Dual-Functional Electronic Devices

Ziao Tian[1,2,#], Borui Xu[1,#], Guangchao Wan[3,#], Xiaomin Han[3], Zengfeng Di[2], Zi Chen[3] & Yongfeng Mei[1,*]

[1] Department of Materials Science, State Key Laboratory of ASIC and Systems, Fudan University, 220 Handan Road, Shanghai 200433, China

[2] State Key Laboratory of Functional Materials for Informatics, Shanghai Institute of Microsystem and Information Technology, Chinese Academy of Sciences, Shanghai 200050, China

[3] Thayer School of Engineering, Dartmouth College, Hanover, 03755 NH, USA

[#] These authors contributed equally to this work

[*] email: yfm@fudan.edu.cn

Supplementary Figures

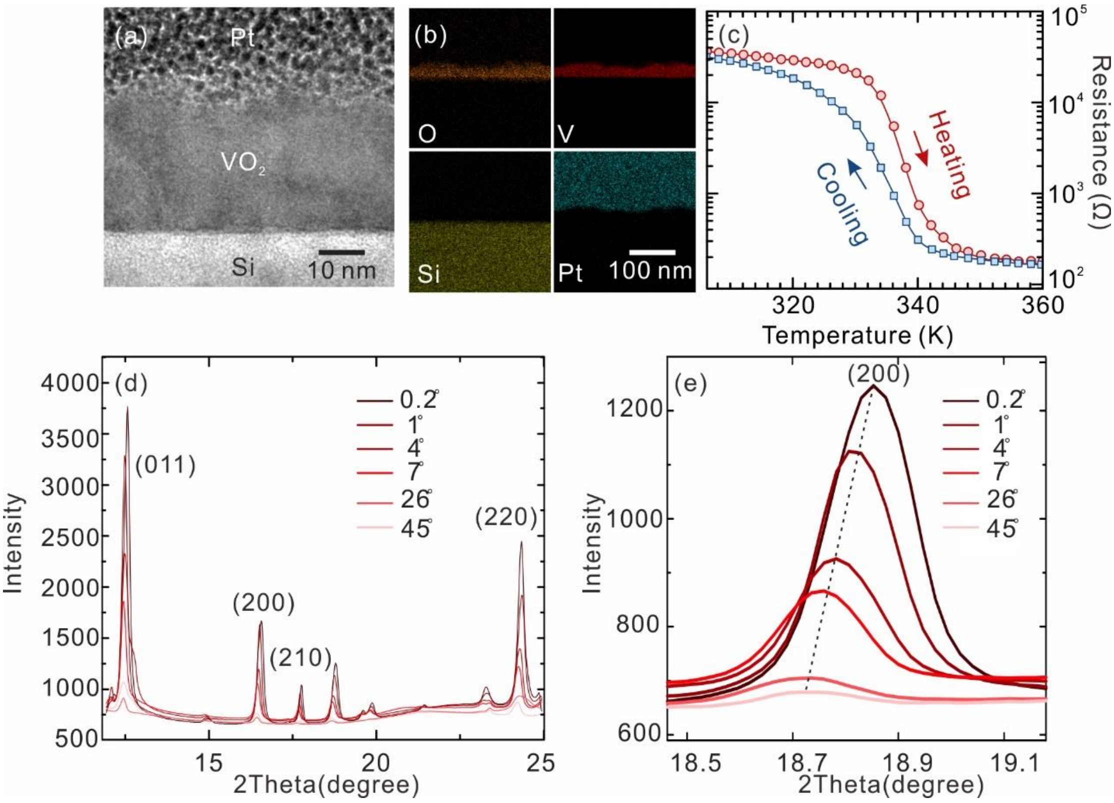

Supplementary Figure 1 | The characteristics of VO_2 nanomembranes. (a) TEM image from the cross section of VO_2 nanomembrane. (b) Elemental mapping showing the distribution of O, V, Si, and Pt. (c) Temperature-dependent resistance of the VO_2 nanomembrane. (d) Grazing incidence X-ray diffraction patterns of VO_2 nanomembrane and (e) corresponding magnified view of (200) peak. The (200) peak position apparently shifts from 18.9 to a lower wavenumber of 18.7 with incidence angle increasing, showing that the strain field near the film surface could be different from that near the film substrate interface. According to previous reports, there is compressive strain in the VO_2 nanomembranes, which agrees with our rolling up behaviors.

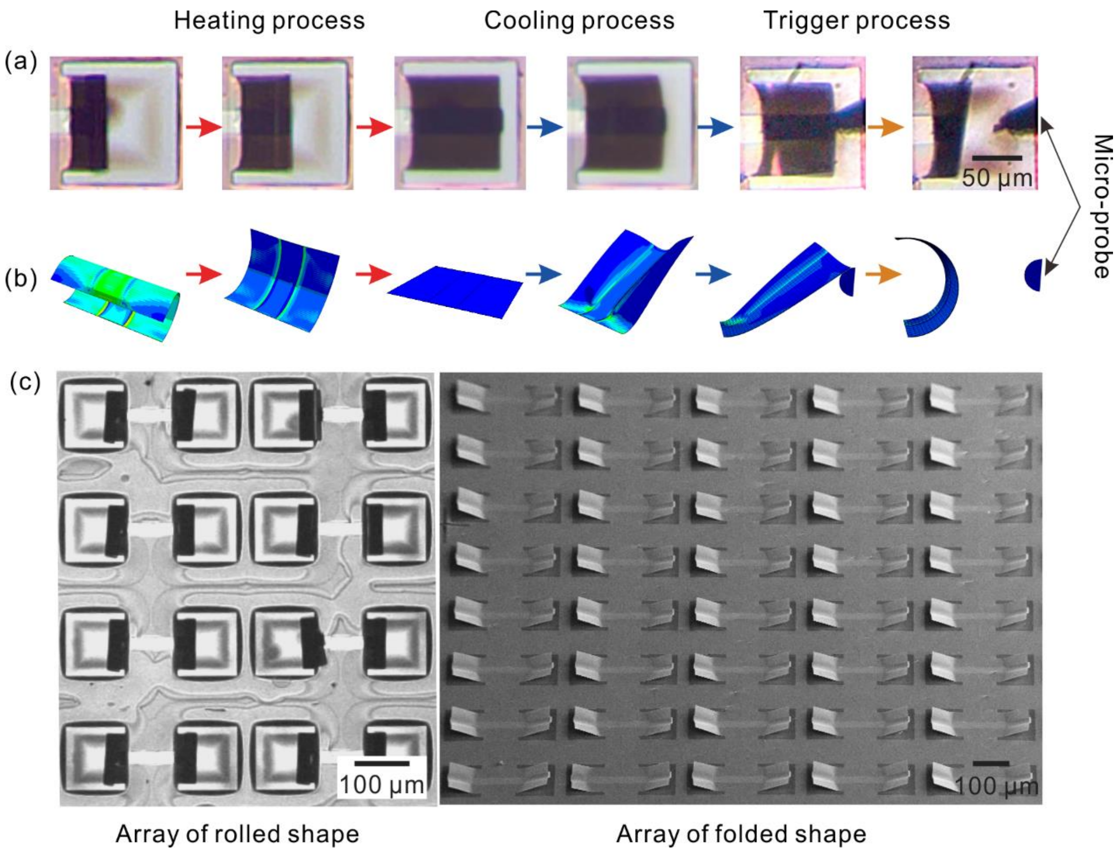

Supplementary Figure 2 | The shape-morphing process of anti-symmetric type and non-volatile shapes. (a) Optical images and (b) FEM results of shape morphing. (c) array of rolled (left) and folded (right) shapes.

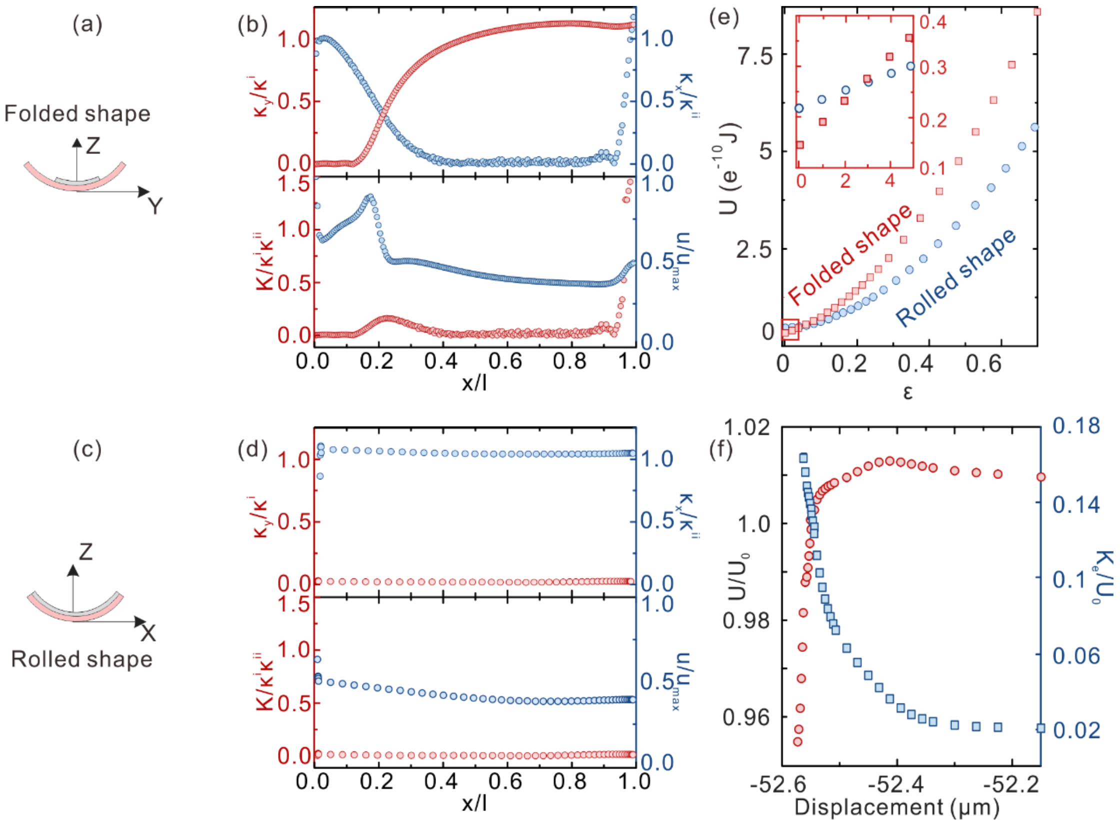

Supplementary Figure 3 | Finite Element Method (FEM) based on ABAQUS. (a) Schematic illustration of the folded shape model. (b) The variations of κ_y/κ^i (top panel, left axis), κ_x/κ^{ii} (top panel, right axis), $K/\kappa^i\kappa^{ii}$ (bottom panel, left axis) and u/u_{max} (bottom panel, right axis, u_{max} is the maximum value of u) versus the x/l in folded shape from FEM. *x/l* is the distance from the clamped edge. (c) Schematic illustration of the rolled shape model. (d) The variations of κ_y/κ^i (top panel, left axis), κ_x/κ^{ii} (top panel, right axis), $K/\kappa^i\kappa^{ii}$ (bottom panel, left axis) and u/u_{max} (bottom panel, right axis, u_{max} is the maximum value of u) versus the x/l in rolled shape from FEM. (e) The elastic energy *U* versus strain for rolled and folled shape. The inset is amplified part in red square. Note that at the initial stage of the strain increasing (ε=0), folded shape has lower strain energy than rolled shape. As a consequence, the

structure tends to turn into folded shape from flat state. As the strain increases beyond 0.029%, the energy of folded shape exceeding rolled shape and finial energy of rolled shape is much lower than folded shape. (f) The variations of the elastic energy U and the kinetic energy K_e of the Cr/VO_2 bilayer during snap-through from FEM. U_0 is the elastic energy of folded shape and d (μm) is the displacement of the probe. Initially, the elastic energy of the thin film U increases due to the external work done by the probe. Snap-through occurs when the probe exceeds the critical position (d≈-52.5μm). During a short period, the elastic energy significantly decreases and gets converted into the kinetic energy, which speeds up the shape transition.

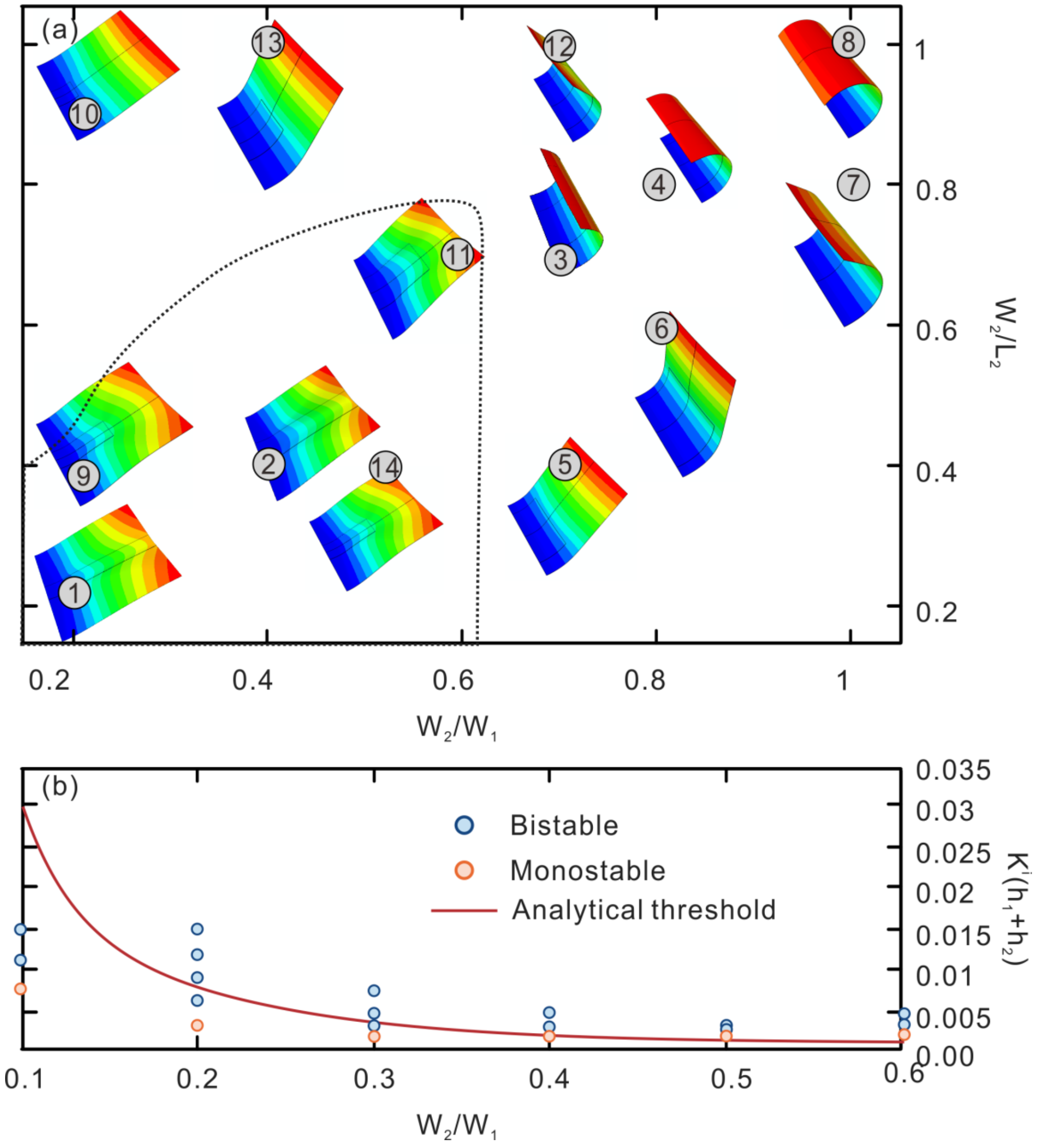

Supplementary Figure 4 | Phase diagram of favorable shape morphing status related to (a) W_2/W_1 vs W_2/L_2 and (b) W_2/W_1 vs K^i. Grey circles are the results from FEM simulations.

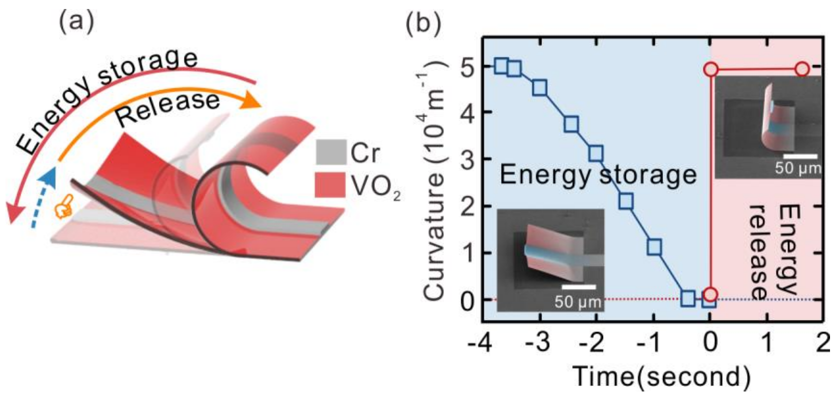

Supplementary Figure 5 | Micro-catapult. (a) The structural and actuating scheme of micro-catapult. The red and blue arrows represent the energy storage process via heating cycle. Orange arrow represents the release process from folded shape to rolled shape. (b) Curvature change of micro-catapult as a function of time. The catapult was triggered at t=0 s. The closure dynamics are characterized by two phases: a slow phase presenting energy storing, a rapid phase presenting energy releasing. Insets show open and closed catapult.

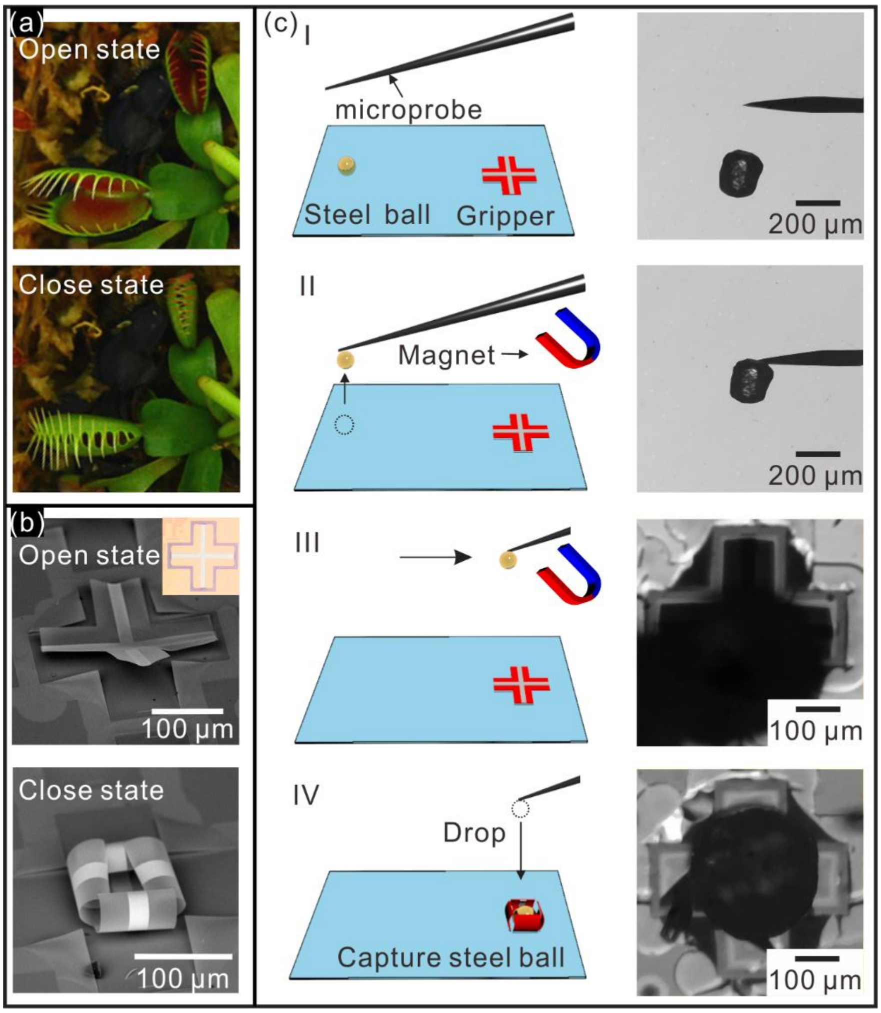

Supplementary Figure 6 | Flytrap-inspired micro-grippers. (a) The *Venus Flytrap* in its open and closed states. (b) micro-grippers with open and closed states. (c) Micro-gripper catching the steel ball. A steel ball with a diameter of 200 μm. The steel ball is attached to microprobe heads under magnetic forces when a magnet is placed near one end of microprobe. The steel ball is moved on the top of the opened micro-gripper. When falling form heights, the steel ball meets the center of the gripper and trigger it.

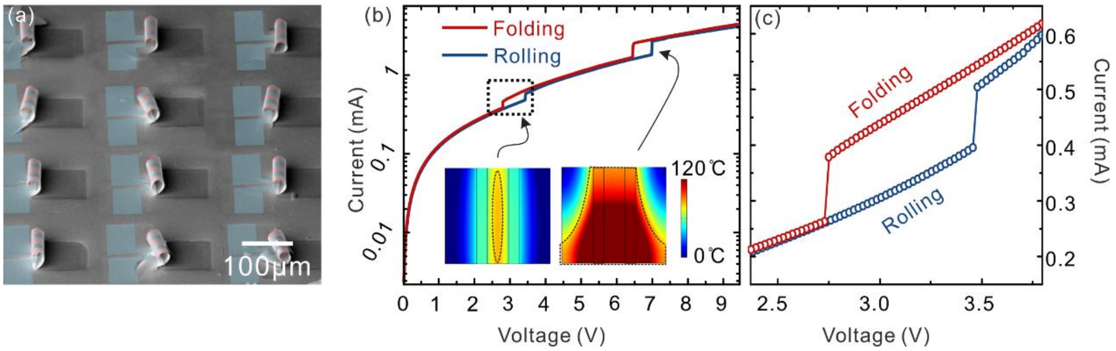

Supplementary Figure 7 | Dual-functional MEMS actuators. (a) An array of Dual-functional MEMS actuators. (b) Experimental current-voltage characteristics of two shapes. Insets show the simulated thermal field profiles corresponding to the two steps on the curves. (c) Amplified figure of current-voltage as the dashed square in (b).

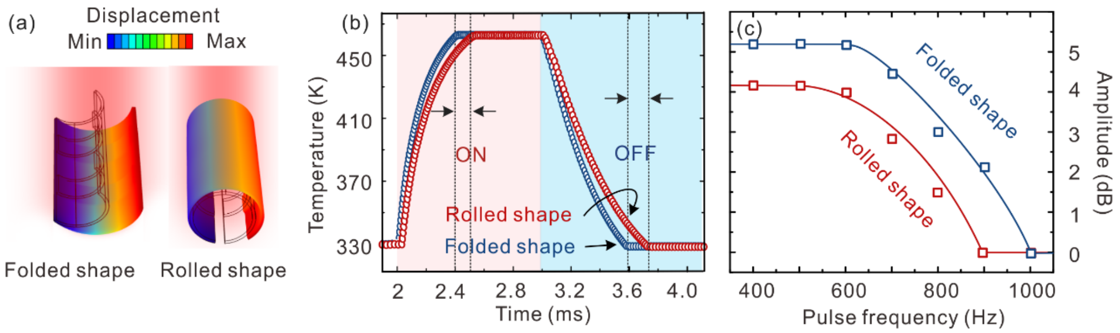

Supplementary Figure 8 | The experimental and simulated response frequencies of both shapes. (a) The simulated displacement of both shapes under laser irradiation. (b) Calculated model results for temperature during a simulated heating pulse. Red region is under heating while blue one is without heating. The ON and OFF tags refer to the difference in time between folded and rolled shape. It is noted that the rise-time and fall-time are different due to the different heat-transfer mechanism. (c) Vibrational amplitude in folded shape and rolled shape as a function of laser pulse frequency.

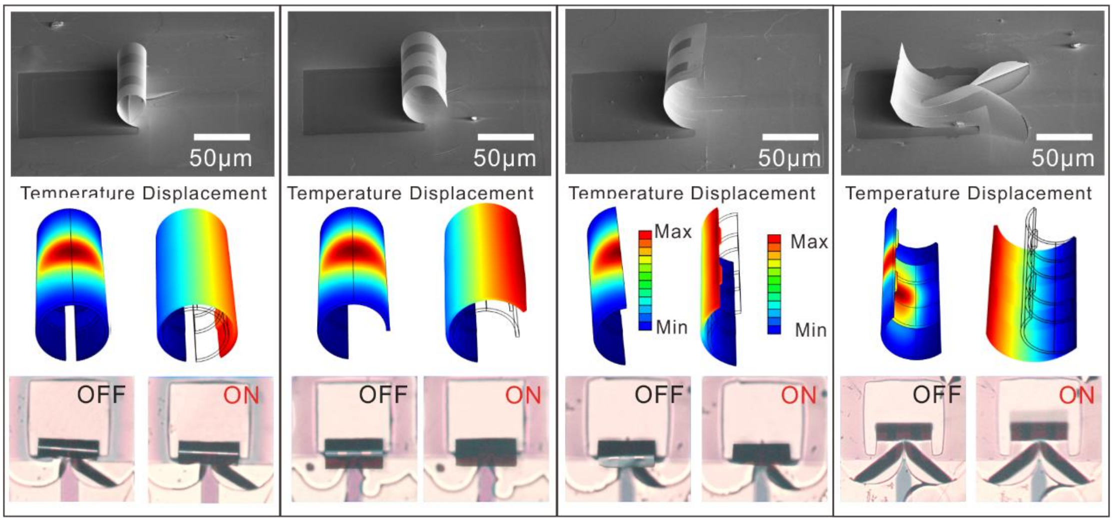

Supplementary Figure 9 | MEMS switches with different curvature. Experimental SEM images are shown in the first row. FEM results are shown in second row. Optical images of switches without (OFF) and with (ON) laser irradiation.

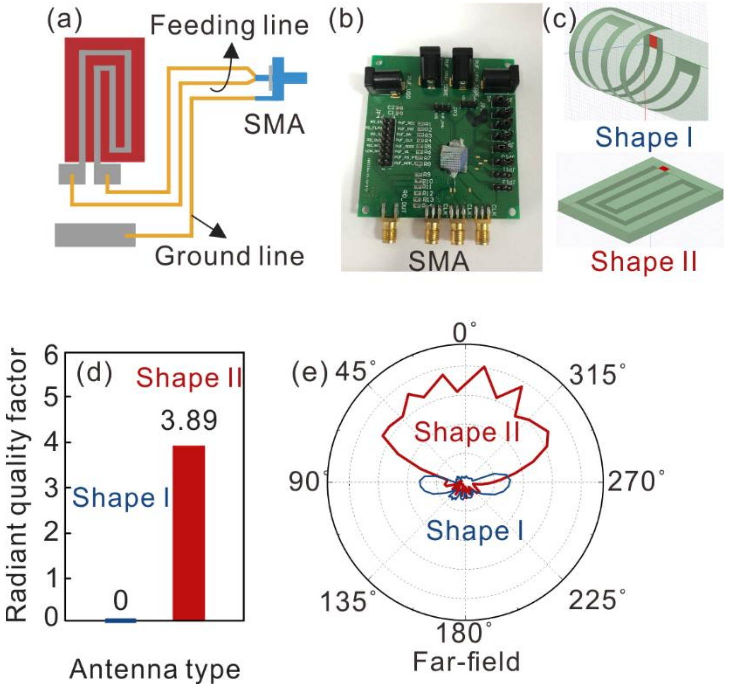

Supplementary Figure 10 | The characteristics of concealable antenna. (a) Schematic illustration of the final 3D configuration of the antenna and the supporting wavy ribbon networks. (b) The photograph of experimental electrical connecting. (c) The FEM models of shape I and shape II. (d) Measured radiant quality factor for two antennas. (e) Simulated radiation patterns (normalized) of the antennas.

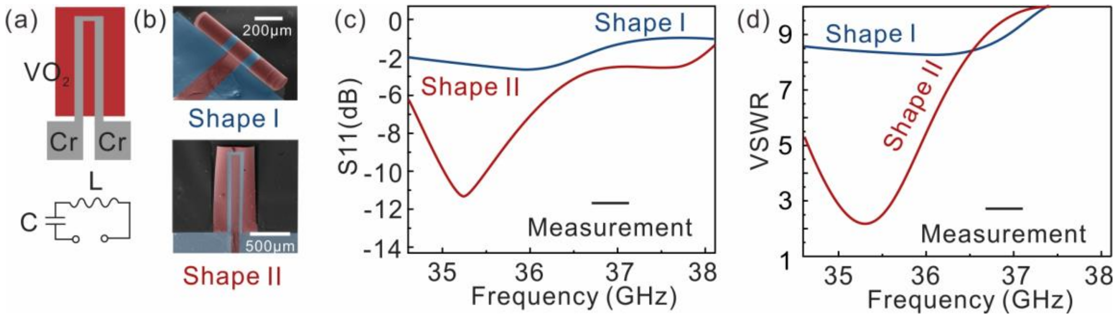

Supplementary Figure 11 | The characteristics of ribbon-like antenna.

(a) Scheme of 2D precursor (top) and equivalent circuit diagrams (bottom) for the device. (b) SEM images of antennas in shape I (top) and II (bottom), respectively. (c) The return loss versus frequency for shape I and shape II. (d) VSWR versus frequency response for two antennas.
