# Supplementary material for: Gaussian-preserved, non-volatile shape morphing in three-dimensional microstructures for dual-functional electronic devices
Source: Nat Commun. 2021 Jan 21;12:509. doi: 10.1038/s41467-020-20843-4 (PMC7820288; doi:10.1038/s41467-020-20843-4)
Supplement: Supplementary file 2 — Description of Additional Supplementary Files [file 41467_2020_20843_MOESM2_ESM.pdf]

Description of Additional Supplementary Files

File Name: Supplementary Movie 1
Description: Experimental Gaussian-preserved shape morphing

File Name: Supplementary Movie 2
Description: Simulated Gaussian-preserved shape morphing

File Name: Supplementary Movie 3
Description: The actuating behavior of rolled shape with various voltages

File Name: Supplementary Movie 4
Description: The first function of MEMS switch based on electrical resistance's change.

File Name: Supplementary Movie 5
Description: The second function of MEMS switch relied on the physical contact.
